# Asynchronous learning: student utilization out of sync with their preference

**DOI:** 10.3402/meo.v21.30587

**Published:** 2016-06-06

**Authors:** Edward K. Lew, Erik K. Nordquist

**Affiliations:** 1 Department of Emergency Medicine, Stanford University, Stanford, CA, USA; 2 Department of Emergency Medicine, John H. Stroger, Jr. Hospital of Cook County, Chicago, IL, USA

**Keywords:** medical education, asynchronous learning, online, medical student, clerkship, curriculum development, emergency medicine, clinical education

## Abstract

**Background:**

Asynchronous learning is gaining popularity. Data are limited regarding this learning method in medical students rotating in emergency medicine (EM). In EM, faculty time is limited to give in-person lectures. The authors sought to create an online curriculum that students could utilize as an additional learning modality.

**Objective:**

The goal was to evaluate effectiveness, participation, and preference for this mode of learning.

**Methods:**

We developed five online, narrated PowerPoint presentations. After orientation, access to the online curriculum was provided to the students, which they could review at their leisure.

**Results:**

One hundred and seven fourth-year medical students participated. They reported the curriculum to be of high quality. Pretest scores were similar for those that viewed all lectures – compliant group (CG) (9.5 [CI 4.8–14.1]) and those that did not view any – non-compliant group (NCG) (9.6 [CI 5.9–13.4]). There was no statistical significant difference in posttest scores between the groups although there was improvement overall: CG 14.6 (CI 6.9–22.1); NCG 11.4 (CI 5.7–17.1). A majority (69.2%) favored inclusion of asynchronous learning, but less than a quarter (22.4%) reported viewing all five modules and more than a third (36.4%) viewed none.

**Conclusion:**

Despite student-expressed preference for an online curriculum, they used the online resource less than expected. This should give pause to educators looking to convert core EM topics to an online format. However, when high-quality online lectures are utilized as a learning tool, this study demonstrates that they had neither a positive nor a negative impact on test scores.

Asynchronous learning and especially online curricula are gaining momentum in medical education. The technique is already frequently used in undergraduate and graduate venues. It provides flexibility, cost-effectiveness, improved resource utilization, and addresses preferences of the millennial generation (1–3). The existing research is primarily focused at the resident level and has shown there is a push to move core conference lectures to asynchronous format (4). As more resident curriculum moves in this direction, medical student education will likely follow. However, little has been published looking at this subject in medical students in emergency medicine (EM).

A recent survey shows that more than half of US medical schools are now requiring rotations in EM in the undergraduate medical curriculum (5). In the same survey, medical student clerkship directors reported a mean of 18 h of lecture time over a 4-week rotation period. A third of that time, full-time EM faculty delivers the didactics and half involve residents (5). In a busy urban emergency department (ED), there is limited faculty time to give monthly in-person traditional lectures to fourth-year medical students (M4s), especially while juggling other administrative, clinical, educational, and research duties. It is increasingly important to explore different avenues of teaching in the EM clerkship, but it is difficult to replace a traditional tried-and-true live lecture series. As such, we sought to create an online curriculum that M4s could utilize as an additional learning modality. The study's objectives were to evaluate effectiveness, participation, and preference for this mode of learning. Current literature is lacking in the EM student clerkship in this regard. We hoped to discover whether it is beneficial to utilize asynchronous modules to either replace or supplement live didactics in this specific population.

## Materials and methods

### Study setting and population

The study site is a tertiary-care, urban, safety-net hospital with more than 127,000 visits annually and home to an Accreditation Council for Graduate Medical Education (ACGME)-accredited Emergency Medicine Residency program. M4s rotate monthly, ranging from those interested in pursuing EM residency to those rotating to complete school requirements or out of pure interest. All M4s were eligible for the study.

### Study curriculum and protocol

We developed an online, narrated lecture series on subjects that were not traditional focuses in our medical student curriculum. In total, five topics were chosen: environmental injury, eye emergencies, obstetrics and gynecology, orthopedics, and toxicology. These topics reflect needs of previously surveyed rotating residents (6), which we believed would also benefit current students. As obstetrics and gynecology is the only core rotation for third-year medical students, most would have not had exposure to these topics from an EM standpoint.

PowerPoint was utilized to build and narrate the lectures, which averaged 35 min each. These lectures were then reviewed and edited by our EM faculty with special interest in those areas. They were then converted to a compatible online streaming video format (mp4) and placed on a password-protected website through VimeoPro.

Based on this lecture series, a 25-question multiple-choice written (5 questions from each topic), paper pretest (Supplementary file 1) was developed and approved by our EM faculty experts as questions that were pertinent to EM knowledge and could be directly answered by watching the online lecture series. On their orientation day, M4s rotating in the ED were given the pretest to this IRB-exempt study. Given ethical concerns of educating medical students in a similar fashion, all students were granted access to the online lectures at the same time. After orientation, every student was emailed the link and password to the lectures. They were instructed to view these at their leisure and to alert us if there were any issues in accessing the modules. Toward the end of the 4-week rotation, they were then given a written, paper posttest (Supplementary file 2), which consisted of the same questions as the pretest, but with the questions and answers rearranged. At this time, they were also given the paper survey (Supplementary file 3) inquiring about usefulness of each module and preference for asynchronous learning.

## Results

We enrolled 107 M4s over the 12-month enrollment period (November 2013–October 2014). All 107 students took the pre and posttests. Overall, students who watched the lectures reported that the modules were of high quality (3.2 on a 4-point Likert scale). Twenty-four students completed all five modules – compliant group (CG). Thirty-nine students did not complete any of the modules – non-compliant group (NCG). Pretest performance was similar between the two groups with the mean number correct: CG 9.5 (CI 4.8–14.1); NCG 9.6 (CI 5.9–13.4). Both groups displayed improvement on the posttest, with no statistical significant difference between them; CG 14.6 (CI 6.9–22.1); NCG 11.4 (CI 5.7–17.1).

**Fig. 1:**
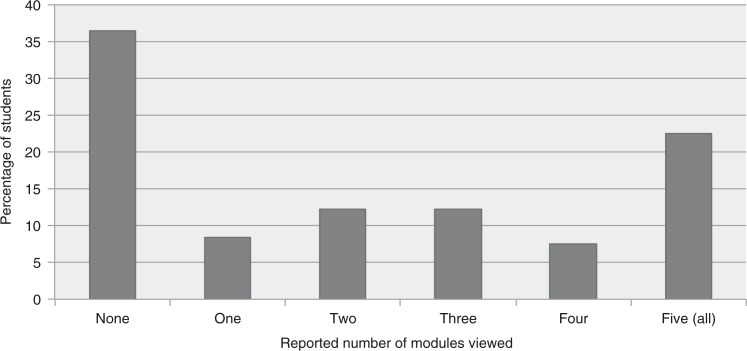
Student utilization of asynchronous curriculum.

All 107 students answered the survey. A majority (69.2%) of the students favored inclusion of asynchronous learning in the curriculum; however, less than a quarter (22.4%) reported viewing all five modules, only 43.0% reported completing at least three, and more than a third (36.4%) viewed none (Fig. 1).

## Discussion

There is a movement to incorporate asynchronous learning in the emergency medicine curriculum, and some have found success using this method in the acquisition of learning (6, 7). Much of this has been directed at the graduate level (residents) however, and lacking in EM students. To the best of our knowledge, only two other studies focused solely on M4s (as ours has) using web-based learning, Baumlin et al. (8) and Jordan et al. (2). Of note, another study, Pusic et al. (9) enrolled medical students as well, but used a stationary computer-aided instruction (PAI) instead of a web-based format. This module had to be done in the ED, not at one's leisure as our curriculum has provided.

Overall, our study should provide pause for EM educators looking to move their curriculum asynchronously in the student clerkship curriculum on two levels: 1) knowledge acquisition, and 2) conflicting learner preference.

1) We found that both the CG and the NCG improved on the posttest. By not viewing any of the modules, the NCG served as a proxy ‘control group’. Both groups had the same access to the web-based lectures, textbooks, traditional lectures, bedside learning, etc.; thus the only difference between the CG and NCG was viewing the modules. The CG had better posttest performance than the NCG. However, this was not statistically significant. This was also reflected by Baumlin (8). Furthermore, in a study of a pediatric asynchronous curriculum in EM, Chang et al. (10) had a small subset of 20 medical students in their asynchronous group that also did not improve in the posttest. Interestingly, Jordan et al. (2) found that medical students did worse on knowledge acquisition using asynchronous curriculum in their university-specific acute care weeklong course. What our study adds in this regard is that in a 4-week long EM-specific clerkship the educational benefits of a newer, modern web-based format should be questioned. This population and setting is more comparable to other institutions that house an EM residency and a concurrent student clerkship (5).

2) Our study did not specifically seek to replace traditional teaching modalities, but 69.2% of our students reported that they favored inclusion of asynchronous learning into our curriculum. Despite this majority, less than half reported viewing at least three modules, and more shockingly 44.8% of students viewed either one or zero. Why do most prefer an online component, but a disappointingly small percentage utilizes it when offered? We could not force our medical students to view these lectures as we found it unethical to link an experimental curriculum with their evaluations and/or grades. This is on par for recent studies (10). Furthermore, we did not want to replace our established curriculum with an unproven online lecture series even though we discovered later that students found our asynchronous format to be of high quality.

More than a decade ago, Baumlin similarly found that their students favored online components to their curriculum. However, only 28% of their population accessed their ‘EMCyberSchool’ (8). In contrast, Jordan et al. (2) found a majority of their students preferred either traditional lectures or remained neutral on the topic. However, this was in a quick 1-week course unique to their medical school curriculum. What this study reveals is that in our modern age, student utilization of an asynchronous format directly contradicts with their preference for the format. Our study specifically shows this with regard to a 4-week EM clerkship, and this is more generalizable to other medical schools.

Although it is true that millennials enjoy technology, and we have shown that they prefer an asynchronous component to their student curriculum, it is unclear how to best deliver it in such a way that educators can guarantee that it will be viewed. One option is to have a designated station to have them watch in the ED while on shift, as seen with Pusic's study (9). However, one would lose the flexibility learners appreciate (2). A solution may be the ‘flipped classroom’ which integrates asynchronous components with in-person modalities (11). It has already found success in medical education areas such as public health, palliative care, and graduate physiology (12–14).

## Limitations

As a novel modality for our students on rotation, we found it unethical not to offer the asynchronous curriculum to everyone at the same time. Fortuitously, some students watched all the modules and some did not watch any, which helped create the CG and NCG group. We could not force everyone to utilize the online lectures without an incentive or punishment, illustrating the difficulty of research involving new educational techniques. In Chang's multicenter study, they also did not enforce participation, and their student subset found similar posttest results (10). As mentioned before, Baumlin found similar results of low participation as our study did (8). Given the comparable findings, we believe our results to be plausible for this population and educational ethical dilemma.

Although the volume of medical students varied month to month, the total sample size is on par with most other studies of online curriculum. Each student has their own motivation for rotating at our ED and we provided similar tools for learning to each of them (e.g., access to faculty, number of shifts, didactics, interactive labs, and online curricula). We assume that everyone had the same opportunities, but how the students utilized each of these was up to them.

Based on our survey, which all participants answered, we were pleased to see that the students who did view the asynchronous curriculum, perceived it as high quality and in a positive light. This likely means the ones that did participate found it valuable. The ones who did not view curriculum did not rank its quality and possibly skewed results. This study calls for investigation on how to best utilize asynchronous curriculum for medical students, as it is clear that they would like access to it.

## Conclusion

M4s rotating through the ED in a 4-week clerkship performed similarly on tests whether they viewed our novel, high-quality, asynchronous curriculum or not. Equally important, despite a majority of students preferring an asynchronous component to their curriculum, when given access to non-required online modules, they utilized it at a less than expected rate. This contemporary study should give pause to educators looking to convert EM topics to an online format in the EM clerkship. Further studies should be done to evaluate a more appropriate way to incorporate an asynchronous curriculum while maintaining appropriate participation rates, as it seems to have similar knowledge acquisition rates. It might also be worthwhile to pursue alternative modalities to integrate online and live components, such as the flipped classroom, for M4s on EM rotations.

## Supplementary Material

Asynchronous learning: student utilization out of sync with their preference

Asynchronous learning: student utilization out of sync with their preference

Asynchronous learning: student utilization out of sync with their preference
