# Supplementary material for: Asynchronous learning: student utilization out of sync with their preference
Source: Med Educ Online. 2016 Jun 6;21:10.3402/meo.v21.30587. doi: 10.3402/meo.v21.30587 (PMC4896965; doi:10.3402/meo.v21.30587)
Supplement: Asynchronous learning: student utilization out of sync with their preference [file MEO-21-30587-s001.docx]

PRETEST

1) When examining the eye with the slit lamp, at what angle do you start positioning the light beam in reference to the eye?

   A. 0 degrees

   B. 30 degrees

   C. 45 degrees

   D. 60 degrees

   E. 90 degrees

2) Approximately, how sensitive is ultrasound for placental abruption?

     A. 10%

     B. 25%

     C. 50%

     D. 75%

     E. 99%

3) Most shoulder dislocations are...

     A. Posterior

     B. Anterior

     C. Lateral

     D. Inferior

     E. Superior

4) What is an example of a muscarnic effect?

     A. Defecation

     B. Dry mucous membranes

     C. Hyperreflexia

     D. Muscle rigidity

     E. Muscle fasciculations

5) When is it considered hypothermia?

    A. Below 35 degrees celsius (95 degrees fahrenheit)

    B. Below 35.5 (95.9)

    C. Below 36 degrees celsius (96.8 degrees fahrenheit)

    D. Below 36.5 (97.7)

    E. Below 37 (98.6)

 6) In evaluating compartment syndrome, above what compartment pressure would a fasciotomy be indicated?

     A. 10

     B. 15

     C. 20

     D. 25

     E. 30

7) Which of these defines an “inevitable abortion?”

A. Os open with no conceptus

     B. Os open with partial conceptus

     C. Os open with normal gestation

     D. Closed os with normal gestation

     E. Closed os with no conceptus

8) What is the average normal intraocular pressure?

    A. 5 mmHg

    B. 10 mmHg

    C. 15 mmHg

    D. 20 mmHg

    E. 25 mmHg

9) What is the minimum percentage TBSA to utitlize Parkland formula for adults?

    A. 10%

    B. 15%

    C. 20%

    D. 25%

    E. 30%

10) Which of these toxins may have the "textbook" smell of garlic?

     A. Cyanide

     B. Arsenic

     C. Methylsalicylate

     D. Nitrile

     E. Zinc phosphide

11) On xray, if you can appreciate an anterior and posterior fat pad sign, what fracture is this suggestive of in adults?

     A. Ulnar

     B. Radial head

     C. Humeral shaft

     D. Trochlea

     E. Olecranon

12) When evaluating for ectopic pregnancy, what is the approximate value of the discriminatory zone?

     A. 1000

     B. 1500

     C. 2500

     D. 3000

     E. 4000

13) When examining the eye, what is the first thing you should do?

     A. Extraocular movements

     B. Visual acuity

     C. Pupillary reflex

     D. Ocular pressure

     E. Visual fields

14) In the managment of heat stroke, what is the goal temperature to maintain the patient below?

    A. 40 celsius

    B. 39 celsius

    C. 38 celsius

    D. 41 celsius

    E. 42 celsius

15) In which situation should activated charcoal be considered?

     A. Two hours after ingestion

     B. Lithium overdose

     C. Non life-threatening overdose

     D. TCA overdose

     E. Iron overdose

16) Which is not a risk factor for adnexal torsion?

     A. Age less than 40

     B. Pregnancy

     C. Ovarian cyst

     D. Ovarian stimulation

     E. Tubal malformations

17) Where is Hutchinson's sign?

     A. Nose

     B. Sclera

     C. Cornea

     D. Forehead

     E. Pupil

18) Which of the following describes a Salter-Harris fracture Type II?

     A. A transverse fracture through the [growth plate](http://en.wikipedia.org/wiki/Growth_plate)

     B. A fracture through all three elements of the bone, the [growth plate](http://en.wikipedia.org/wiki/Growth_plate), [metaphysis](http://en.wikipedia.org/wiki/Metaphysis), and [epiphysis](http://en.wikipedia.org/wiki/Epiphysis)

     C. A compression fracture of the [growth plate](http://en.wikipedia.org/wiki/Growth_plate) (resulting in a decrease in the perceived space

between the [epiphysis](http://en.wikipedia.org/wiki/Epiphysis) and [diaphysis](http://en.wikipedia.org/wiki/Diaphysis) on x-ray

     D. A fracture through the growth plate and the [metaphysis](http://en.wikipedia.org/wiki/Metaphysis), sparing the [epiphysis](http://en.wikipedia.org/wiki/Epiphysis)

     E. A fracture through [growth plate](http://en.wikipedia.org/wiki/Growth_plate) and [epiphysis](http://en.wikipedia.org/wiki/Epiphysis), sparing the [metaphysis](http://en.wikipedia.org/wiki/Metaphysis):

19) Which is a common finding in the sympathomimetic toxidrome?

     A. Dystonia

     B. Lacrimation

     C. Urinary retention

     D. Bradycardia

     E. Mydriasis

20) Blood vessel damage caused by electricity resembles what injury?

    A. Crush

    B. Thermal

    C. Freezing

    D. Chemical

    E. Penetrating

21)  What is chemosis?

    A. Blood in anterior chamber

    B. White cells in anterior chamber

    C. Inflammation beneath conjunctival layer

    D. Bleeding beneath conjunctival layer

    E. White blood cells in aqueous humor

22.) A mallet finger is...

     A. Caused by forced extension of a flexed finger

     B. Caused by damage to the flexor tendon

     C. Caused by forced flexion of extended finger

     D. Caused by rheumatoid arthritis

     E. Caused by a fracture of the distal phalange

23) Overdose of mescaline may give what symptoms?

     A. Sedative-hypnotic

     B. Anticholinergic

     C. Cholinergic

     D. Hallucinogenic

     E. Sympathomimetic

24) What is the most common cause of vaginitis?

     A. Bacterial vaginosis

     B. Candida

     C. Trichomonas

     D. Chemical

     E. Atrophic

25) Which is not correct in the management of frostbite?

   A. Pain control

   B. Rubbing skin to assist in rewarming

   C. Debriding blisters

   D. Wet rewarming

   E. Immobilizing extremity
