# Supplementary material for: Asynchronous learning: student utilization out of sync with their preference
Source: Med Educ Online. 2016 Jun 6;21:10.3402/meo.v21.30587. doi: 10.3402/meo.v21.30587 (PMC4896965; doi:10.3402/meo.v21.30587)
Supplement: Asynchronous learning: student utilization out of sync with their preference [file MEO-21-30587-s003.docx]

**Fourth-year medical student survey on online curriculum**

***1) How useful to your learning did you find the following online emergency medicine lectures? (please circle ‘N/A’ if did not view lecture)***

|  | N/A, did not view | Not useful at all |  |  | Extremely useful |
| --- | --- | --- | --- | --- | --- |
| Toxicology | N/A | 1 | 2 | 3 | 4 |
| Environmental | N/A | 1 | 2 | 3 | 4 |
| Orthopedics | N/A | 1 | 2 | 3 | 4 |
| Eye | N/A | 1 | 2 | 3 | 4 |
| OBGYN | N/A | 1 | 2 | 3 | 4 |

***2) Would you prefer live in-person lectures, online lectures, or a mixture of both? (please circle)***

In-person Online Mixture of both
